# Ultrasonic Vocalizations Induced by Sex and Amphetamine in M2, M4, M5 Muscarinic and D2 Dopamine Receptor Knockout Mice

**DOI:** 10.1371/journal.pone.0001893

**Published:** 2008-04-02

**Authors:** Haoran Wang, Shuyin Liang, Jeffrey Burgdorf, Jurgen Wess, John Yeomans

**Affiliations:** 1 Department of Psychology, Center for Biological Timing and Cognition (CTBC), University of Toronto, Toronto, Canada; 2 Falk Center for Molecular Therapeutics, Northwestern University, Evanston, Illinois, United States of America; 3 Molecular Signaling, Laboratory of Bioorganic Chemistry, National Institute of Diabetes and Digestive and Kidney Diseases (NIDDK), National Institutes of Health, Bethesda, Maryland, United States of America; James Cook University, Australia

## Abstract

Adult mice communicate by emitting ultrasonic vocalizations (USVs) during the appetitive phases of sexual behavior. However, little is known about the genes important in controlling call production. Here, we study the induction and regulation of USVs in muscarinic and dopaminergic receptor knockout (KO) mice as well as wild-type controls during sexual behavior. Female mouse urine, but not female rat or human urine, induced USVs in male mice, whereas male urine did not induce USVs in females. Direct contact of males with females is required for eliciting high level of USVs in males. USVs (25 to120 kHz) were emitted only by males, suggesting positive state; however human-audible squeaks were produced only by females, implying negative state during male-female pairing. USVs were divided into flat and frequency-modulated calls. Male USVs often changed from continuous to broken frequency-modulated calls after initiation of mounting. In M2 KO mice, USVs were lost in about 70–80% of the mice, correlating with a loss of sexual interaction. In M5 KO mice, mean USVs were reduced by almost 80% even though sexual interaction was vigorous. In D2 KOs, the duration of USVs was extended by 20%. In M4 KOs, no significant differences were observed. Amphetamine dose-dependently induced USVs in wild-type males (most at 0.5 mg/kg i.p.), but did not elicit USVs in M5 KO or female mice. These studies suggest that M2 and M5 muscarinic receptors are needed for male USV production during male-female interactions, likely via their roles in dopamine activation. These findings are important for the understanding of the neural substrates for positive affect.

## Introduction

Reproduction requires detecting, recognizing and courting a potential mate in mammals. Progress through these stages is guided by cues involving a wide range of sensory systems [1]. In mice, the vomeronasal organ is thought to be the primary sensory system responsible for the detection of sexual attractants, which regulate a variety of responses including mate recognition [2]. Urine and related pheromones are important inducers of these behaviors [2], [3]. Individual recognition is not only a key component of behaviors that are vital for reproductive success [3], but also crucial for social behavior. This recognition is known to be often mediated by olfactory cues in rodents, but how male-female mutual recognition occurs and works for behaviors still remains controversial [4]–[6].

Almost half a century ago, several rodents, including mice, were discovered to produce USVs [7], [8]. Mice not only can emit USVs, but also can perceive USVs [9]. They communicate both in the human-audible range with squeaks for long-distance warnings [10], and in the ultrasound range for short-distance communication [11], [12]. Mouse USVs can be induced by urine and pheromones [1], [13], [14], and are often observed during sexual behavior [13], [15]. USVs may be helpful for sexual behavior [16], [17]. In mouse pups, production of USVs is affected by maternal isolation, cold, rotation and genotype [18]. Mouse USV frequencies are especially variable and complex [12], [13], [15], [19]. Mouse USVs elicited by female urine possess many characteristics of song, consisting of several different syllable types, whose temporal sequencing includes the utterance of repeated phrases [14]. When male mice encounter female mice or their pheromones, they emit USVs with frequencies ranging from 25–120 kHz [1], [13], [14].

Cholinergic systems play important roles in modulating functions of dopaminergic systems in the brain [20], [21]. Muscarinic receptors play essential roles in brain dopamine release, reward, and drug dependence [20]–[25]. M5 muscarinic receptors are preferentially expressed on tegmental dopaminergic neurons, whereas M2 muscarinic receptors are widely expressed, especially on cholinergic cells and terminals [24], [26]–[29]. Dopamine is the best-defined neurotransmitter activated by rewards, and is needed in a variety of reward functions including emotion, positive reinforcement, food intake and sexual behavior [23], [30]–[33]. In rats, 50-kHz USVs were proposed to reflect positive affective states, whereas 22-kHz USV were shown to be an index of negative states [34]. In mice, USVs may play an important role in social reward [12], [16] or social memory [35]. However the emotional and behavioral importance of USVs in mice is unclear [36].

A few genes have been found involved in controlling USV production in mice. CaMKIV contributed to the injury and fear-induced vocalizations in adult mice [19]. Disruption of a single copy of FOXP2 gene, which is mutated in a monogenic form of speech and language impairment in humans [37] and is also associated with schizophrenia [38], reduced USVs in response to maternal separation in mice [39]. Moreover, µ-opioid receptor knockout mouse pups emitted fewer ultrasonic vocalizations when removed from their mothers, indicating a deficit in attachment behaviors [40]. Recently, MeCP2 [41], oxytocin [42], vasopressin [43], serotonin [44], and the endocanabinoid system [45] were also shown to play a role in USV production. In spite of these findings, molecular and genetic influences on USV production are largely undetermined.

This study was undertaken to: 1) investigate mouse USV induction and its biological significance; 2) characterize sexually elicited USVs in mice during male-female interaction; 3) examine the effects of M2, M4 and M5 muscarinic and D2 dopamine receptor gene deletion and the dopamine activator amphetamine on USV production. We found M2 and M5 muscarinic, but not M4 and D2 receptors, play crucial roles in the male-emitted USVs. These findings provide a new approach for exploring male-female recognition, communication, affective states, and sexual reward in mice.

## Results

### Urine-induced USVs in mice (Experiment, Exp. 1)

**Figure 1:**
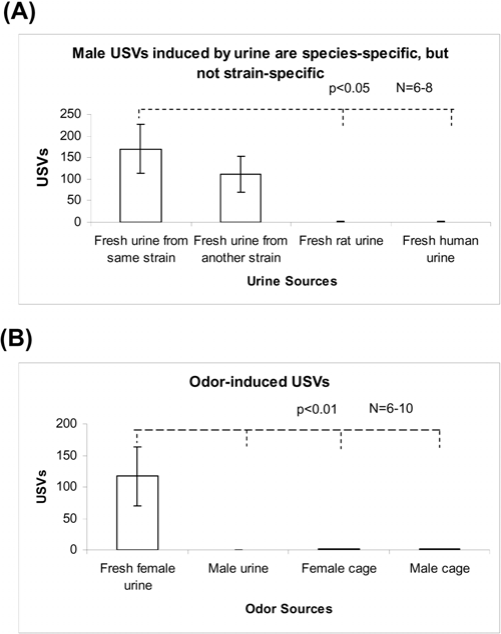
Urine-induced USVs in mice were species-specific, but not strain-specific in Exp. 1. (A) Fresh female mouse urine induced USVs in male mice, fresh female rat urine or human urine did not induce USVs in male mice. C57BL/6 mouse urine similarly induced USV in CD1 x 129 (C57–C129), vice versa (data not shown). (B) Dirty female cages induced a few USVs in male mice, and male mouse urine (second column) did not induce USVs in female mice.

Female mouse urine with its pheromones can induce USVs in male mice [46]. Can female rat or human urine induce USVs in male mice, and can male urine induce female USVs? As shown in Figure 1A, fresh female urine induced high levels of USVs in male mice. No USV was observed 5 min after urine addition (Table S1). Female C57BL/6 mouse urine induced USVs in CD1x129 male mice (Figure 1A), and CD1x129 female mouse urine induced USVs in C57BL/6 male mice too (data not shown). However, fresh female rat or human urine rarely induced USVs in male mice (Figure 1A). In addition, male fresh urine did not induce female USVs, and dirty cages had little effect (Figure 1B). These results suggest that urine-induced USVs in mice are species-specific, but not strain-specific, and that urine-induced USVs are unidirectional: from female urine to male USVs, but not vice versa.

### Direct contact of male with female, but not odor or sight of the female, induced USVs in males (Exp. 2)

**Figure 2:**
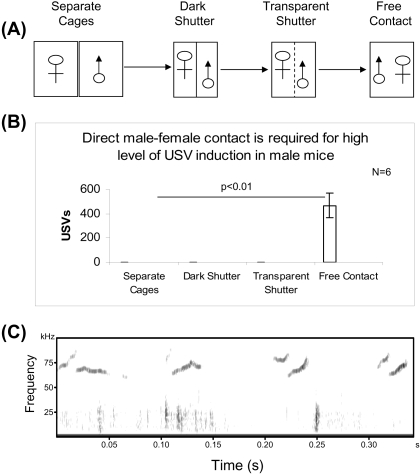
Direct contact of males with females induced high levels of USV production in males, while odor and sight of the whole female mice body did not in Exp. 2. (A) Schematic representation of the experimental procedures. The experiments were divided into 4 stages. They are separated by cages, dark shutter, transparent shutter, and no shutter (free contact), respectively. (B) No USVs were detected when male and female were separated in two cages or in one cage but separated by a dark shutter. A few USVs were detected once the dark shutter was replaced by a transparent shutter. Full USV production was observed when the shutter was removed and male and female mice were free for direct contact. (C) USV burst out after two mice had direct contact. Representative USVs are shown.

Although fresh female urine or pheromones can induce USVs in male mice [14], the roles of odor, sight of the female body, or male-female tactile contact remain unknown. Therefore, as shown in Figure 2A, we tested male USVs after female exposure in 4 ways. No USVs were detected when male and female were separated in two different cages even if the two cages were placed tightly together (Figure 2B). When the male mouse was placed into the female cage but separated by a dark shutter (Figure 2B), no USVs were recorded. In 2 of 6 pairs, a few USVs were detected once the dark shutter was switched into a transparent shutter (Figure 2B). Full numbers of USVs were not observed until the shutter was removed, and then male and female mice were free to contact one another (Figure 2B). USVs were recorded soon after direct male-female contact (Figure 2C). These results showed that direct contact of male with female, but not odor or sight of the female body, is sufficient for inducing high levels of USVs in males.

### Males emitted complex USVs during male-female interaction, while females emitted only squeaks (Exp. 2–5)

**Figure 3:**
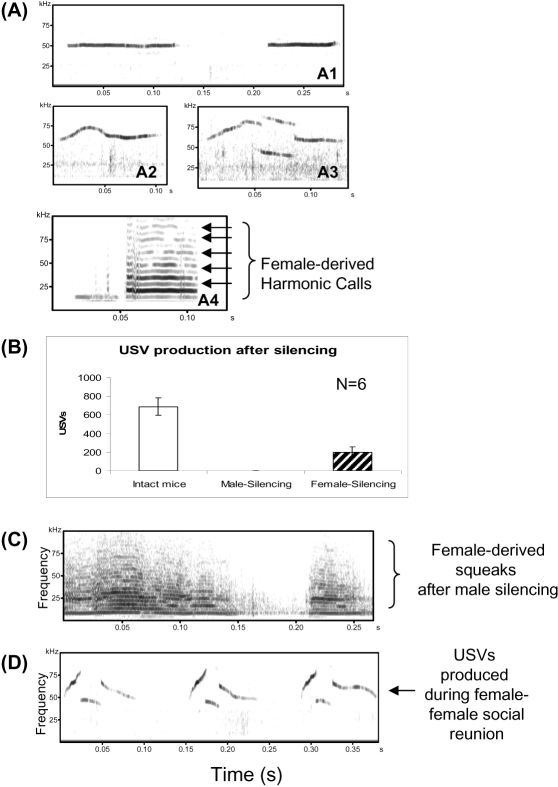
Males and females call differently during male-female interaction in Exp. 2, 4 and 5. (A) Representative sonograms of mouse ultrasonic vocalizations produced during male-female interaction. 4A1: flat USV, 4A2: frequency-modulated sine-wave-like USV and 4A3: broken USV, and 4A4: breached-harmonic USV. 4A5: human audible dense-layered squeaks. (B) No USVs were detected when male were silenced by simple mouth taping; but when females were silenced, many USVs were detected, suggesting that USVs recorded during male-female interaction were emitted from the male. (C) After aggressive attacks from male mice, females produced a few squeaks, observed on sonograms as harmonic dense-layered calls. (D) When reunited with another female after 4 hour's separation as described before [73], female mice emitted frequency-modulated USVs as those produced by male mice during male-female interaction. Three representative broken USVs were shown.

Considering the influence of different genetic backgrounds of the mice and other inducing factors on the patterns of calls [1], [13], [14], [47], 3 representative sonograms of mouse (CD1x129) USVs obtained during male-female interaction are shown in Figure 3A. These are flat USVs (bandwidth ≤5-kHz) (Figure 3A1), frequency-modulated sine-wave-like USVs (Figure 3A2, bandwidth >5-kHz), and broken USVs (Figure 3A3), respectively. A representative squeak is also shown (Figure 3A4).

To further clarify the origin of regular USVs and squeaks produced during male-female interaction, we performed silencing tests in which one partner was silenced by closely taping its mouth, while the other partner was allowed to call freely. As a result, in pairs when females were silenced, male mice still produced many USVs (Figure 3B), but no squeaks (data not shown); however except squeaks (Figure 3C), no USVs were detected when males were silenced (Figure 3B and Table S2). These results strongly suggest that, during male-female interaction, males and females call differently: males call in USVs; and females call in squeaks.

Furthermore, two independent USVs have never been found to overlap in at least 100 male-female interactions; however, in our female-female social reunion studies, two USVs were frequently found to overlap (data not shown). These data indicate that only one partner emitted USVs during male-female interaction, and this USV caller is the male. In many cases, USVs did overlap with “squeaks” during male-female sexual interaction, especially during mounting (Figure S1). Considering the results obtained in silencing studies and the results described above, it is the female apparently that produced squeaks during male-female interaction.

In addition, regarding the relationship between mouse calls and affective states, we found that squeaks were often detected when the female was sexually attacked by the male, during which the female ran away from or fought off the male. Squeaks (Figure 3C and S1) were especially detected when the male mounted the female for copulation and the female resisted (video data not shown). No squeaks were detected without pairing male and female or when females showed no signs of being annoyed or resisting forced sex or other aversive attacks during male-female interaction. However, females did produce USVs when two females reunited for social play after hours of separation (Figure 3D). Males produced USVs in response to fresh female urine (Figure 1), female contact (Figure 2), or in response to amphetamine (see “amphetamine-induced USVs” below) which induce positive states, but males did not produce USVs in response to males regardless of whether they displayed vigorous aggressive behaviour or not (data not shown).

Taken together, these data showed that male and female calls differed depending on affective consequences. During male-female interaction, males called in USVs indexing positive states that are associated with approach behaviors; but females called in the squeaks indexing negative states that are associated with avoidance behavior [10].

### Pairing of males with ovariectomized females did not alter male's USVs (Exp. 3)

**Figure 4:**
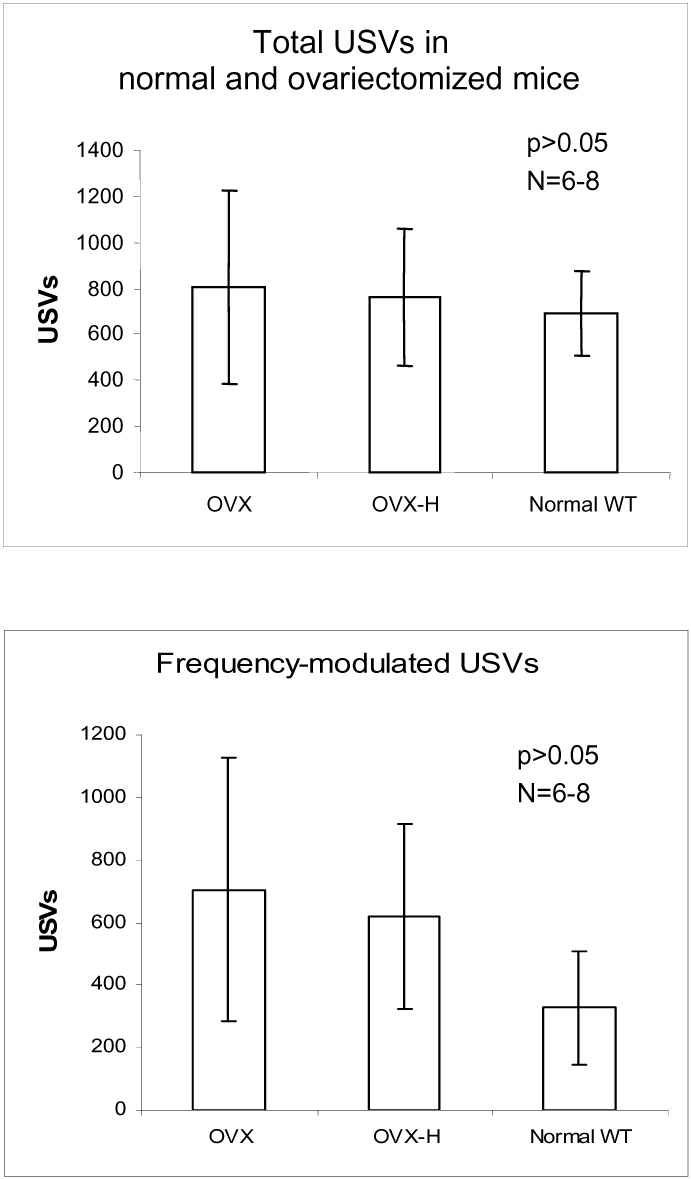
Female sexual hormone status did not affect USVs of male mice when interacting with females in Exp. 3. Ovariectomy in adult female did not affect males' USVs. OVX: female mice were paired with male mice 21 days after ovariectomy. OVX-H: like OVX, but the mice received hormone replacement (three injections, 2 of estrogen first and followed by 1 of progesterone) on three consecutive days. WT: untreated wild-type female mice. Upper panel: total USVs; bottom panel: frequency-modulated USVs. There were slightly more male USVs to both groups of OVX female mice.

To study the role of female sexual hormones in male USVs, we performed bilateral ovariectomy. Vaginal smears after behaviour tests and biopsy post mortem were examined to confirm the completeness of the surgeries. Ovariectomies in adult females, tested 14 or 21 days after surgery, neither affected the total USVs of males (Figure 4, upper panel) nor frequency-modulated USVs (Figure 4, bottom panel). This suggests that ovariectomy or ovarian status did not change the adult female's ability to induce male's USV, and that the gonadal hormone status of the female mice did not significantly affect male-female associated USVs of male mice. This allows use of naïve intact adult females to pair with males to study USVs [48].

### Coincidence of mounting behaviour and production of USVs by males and squeaks by females (Exp. 4)

**Figure 5:**
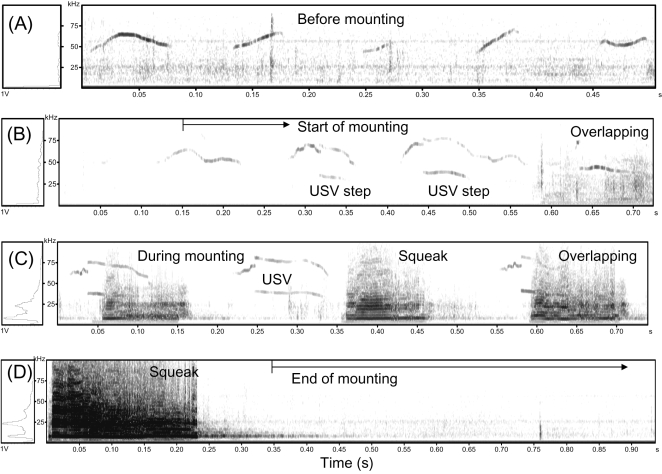
Coincidence of mounting behaviour and production of USVs by males and squeaks by females in Exp. 4. Coincidence of mounting behaviour and production of USVs and squeaks were analyzed by comparing the sonograms of mouse calls before, during, and after mounting behavior with simultaneously recorded videos as described in Materials and Methods. (A) Representative USVs emitted by male mice before mounting. (B) Male USVs shifted from “sine” to “step-like” USVs after initiation of male- mounting-female. (C) Coincidence of frequency-modulated “step-like” USVs emitted by male mice and squeaks produced by female mice during mounting/intromission. (D) No call was detected within about 10 sec after the end of mounting (full data not shown).

As described above, males called in USVs and females called in squeaks during male-female interaction. To study this more carefully, we investigated the coincidence of sexual behaviour (e.g., mounting) and production of USVs and squeaks. Before mounting, males usually called in either flat or continuous frequency-modulated USVs (Figure 5A). However, when males mounted females, males shifted to frequency-modulated broken “step-like” USVs with 70-kHz and 40/80 harmonic frequencies until the end of mounting (Figure 5B and C). Meanwhile, females called in squeaks which often overlapped with USVs (Figure 5C). These calls lasted as long as 20 sec. At the end of mounting, there usually followed an approximately 10 sec (10.66±2.58 sec, mean±SD) period of call-absence (Figure 5D). These results indicate a dynamic change of male USVs and an occurrence of female squeaks during mounting behavior.

### Reduced male USVs in M5 muscarinic receptor KO mice (Exp. 4)

**Figure 6:**
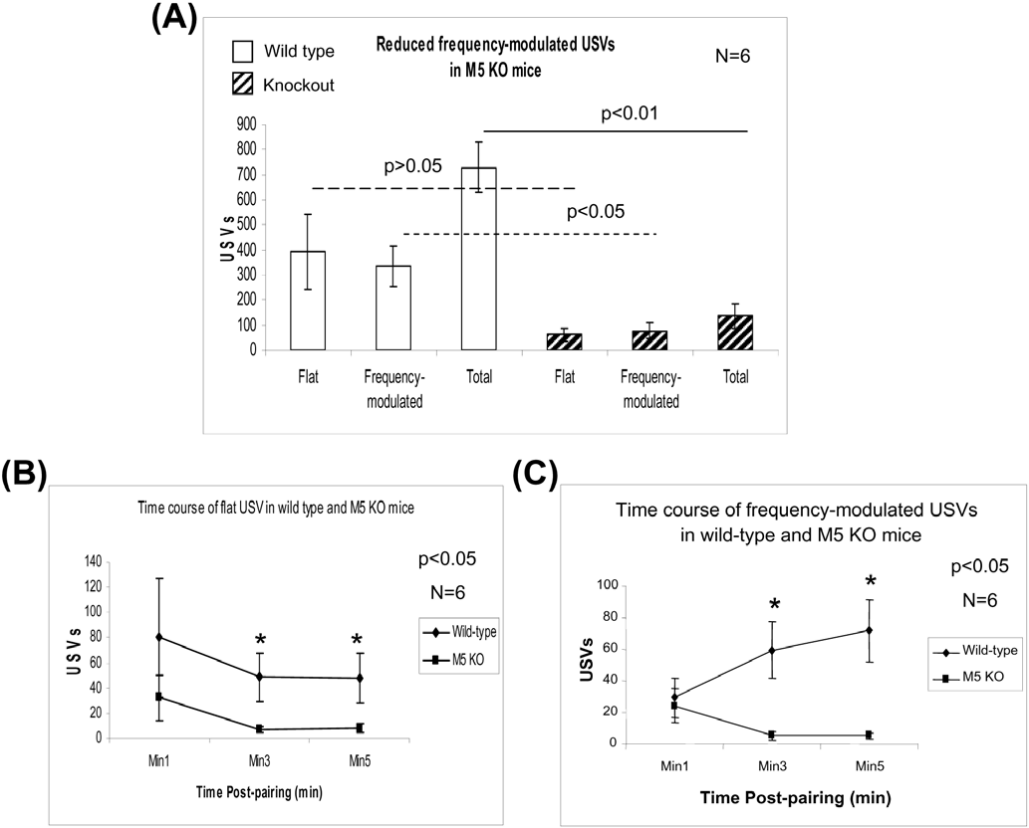
Reduced male USVs in M5 KO mice in Exp. 4. (A) Both the total and frequency-modulated USVs (Bandwidth >5-kHz) were reduced in M5 KO mice of the CD1x129 strain. A smaller, non-significant reduction was seen in flat USVs. Each bar shows the mean number of USVs in each condition. (B) Time courses of flat USVs in wild-type and M5 KO mice. Flat USVs in M5 KO mice were reduced at min 3 and 5. (C) Time courses of frequency-modulated USVs in wild-type and M5 KO mice. Frequency-modulated USVs were also reduced at min 3 and 5. The white and the dashed bars represent wild-type and knockout mice, respectively.

The M5 receptor is required for prolonged dopamine release in nucleus accumbens following mesopontine stimulation in mice [23], [25], and is also important for brain-stimulation reward in rats [23]. The M5 receptor is involved in opiate reward [22], latent inhibition learning and amphetamine-induced locomotion [28]. To investigate the effects of the M5 receptor in USV production, we studied USVs during male-female interaction using M5 KO mice (CD1x129 strain). The total number of frequency-modulated USVs was reduced by almost 80% in M5 KO mice (Figure 6A, p<0.01). The reduction in flat USVs was not significant compared with wild-type mice (p>0.05). The time-course analysis of USVs showed that both total USVs and frequency-modulated USVs decreased in a time-dependent manner in M5 KO mice. Almost no USVs were detected in M5 KO mice 3 min after male-female pairing (Figure 6B and C). On the other hand, USV production remained high in wild-type mice (Figure 6B and C). Therefore, M5 muscarinic receptors play an important role in modulating male-female interaction associated USVs, especially frequency-modulated USVs in mice.

### USVs were reduced in M2 KO mice, but not in M4 and D2 KO mice (Exp. 4)

Alterations in cholinergic functions are associated with cognitive and behavioral symptoms [49]. Hippocampal acetylcholine neurotransmission was dysregulated and cognition was impaired in M2, M4 and M2/M4 muscarinic receptor knockout mice [50]. Both M2 and M4 muscarinic receptors participate in dopamine modulation in central nervous system [21], [24].

**Figure 7:**
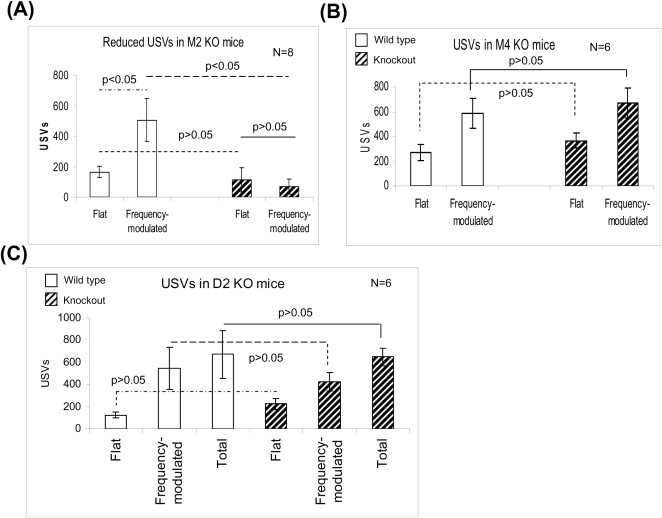
USVs were lost in most of the M2 male KO mice tested, but not in M4 KO and D2 mice in Exp. 4. (A) Frequency-modulated USVs were markedly reduced in M2 KO mice during male-female interactions. (B) Frequency-modulated USVs were not changed in M4 KO mice. (D) No frequency-modulated or total USV changes were detected between wild-type and D2 KO male mice. The white and the dashed bars represent wild-type and knockout mice, respectively.

To examine the roles of M2, M4 and D2 receptors in male-female interactions, we measured USVs of M2 and M4 KO mice. USVs were not detected in 6 out of 8 M2 KO mice (Figure 7A and Table S3) and the loss of USVs in male mice was associated with a corresponding loss of male-chasing-female, male-sniffing-female and male-mounting-female behaviors. On the other hand, in M4 KO mice, frequency-modulated USVs remained unchanged, with a small increase in flat USVs (p>0.05, Figure 7B). D2 dopamine receptors are widely involved in reward, motivation, emotion and cognition. USVs were not changed in the D2 KO mice (Figure 7C, p>0.05). These results suggest that M2 receptors play an important role in male USVs and sexual behavior in response to female mice, but M4 and D2 receptors are less critical, at least in adult mice during male-female interaction.

### Amphetamine induced USVs in males, but not females or M5 KO mice (Exp. 5)

**Figure 8:**
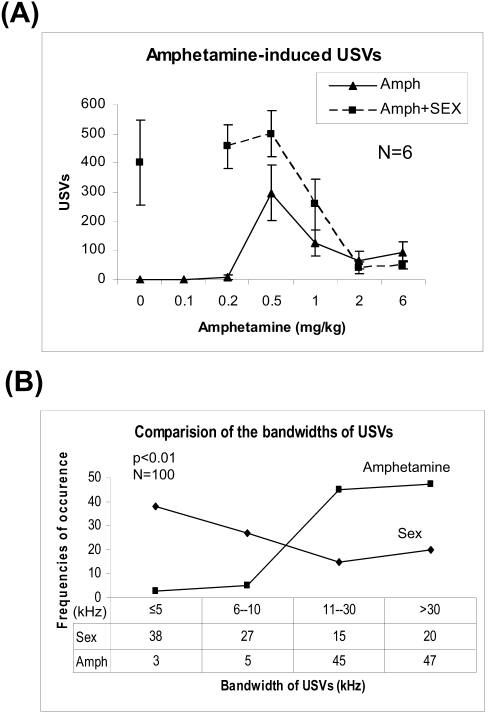
Amphetamine administration dose-dependently stimulated USV production in isolated male mice, and altered the USVs produced during male-female interaction in Exp. 5. (A) D-amphetamine sulfate was given to the mice at 0.1, 0.2, 0.5, 1.0, 2.0 and 6 mg/kg (i.p.), respectively. Amphetamine only stimulated spontaneous USV production in male wild-type mice (CD1x129) (triangles). D-amphetamine sulfate (0.2, 0.5, 1.0, 2.0, 6 mg/kg, i.p.) did not induce USVs in isolated female or M5 KO mice (since no USV was induced, related data were omitted). As compared with saline injection on test day 1, during male-female interaction, male mice produced fewer USVs after 2 or 6 mg/kg amphetamine injection (i.p.) in males (squares, p<0.05). (B) Comparison of bandwidths of sex- and amphetamine-induced USVs in males. One hundred of USVs were randomly selected from large scale of USVs induced by sex and amphetamine (5 mg/kg), respectively. Bandwidths were obtained by measuring the upper frequencies and lower frequencies with Avisoft SASLab Pro software according to the instruction. Amphetamine apparently induced more frequency-modulated USVs (bandwidth >5-kHz) than sex (p<0.01, χ-test). Amph = amphetamine.

The dopamine agonist, d-amphetamine sulfate (Sigma, USA) induced vigorous USVs in isolated male wild-type mice (CD1x129) at 0.5 mg/kg and the effects became weaker as doses increased from 1.0 to 6 mg/kg (Figure 8A). As shown in Figure 8B, in response to amphetamine induction, flat USVs (bandwidth ≤5-kHz) of males accounted for only 3% of the total. Therefore, more frequency-modulated USVs (97% in total, bandwidth >5-kHz) were induced by amphetamine (0.5 mg/kg) than by sex (62% in total, p<0.01). Amphetamine (0.2, 0.5, 1.0, 2.0, 6.0 mg/kg, i.p.) did not induce USVs in isolated female mice or M5 KO male or female mice. These results suggest that the M5 receptor gene is required for amphetamine-induced USVs, and that the effects of amphetamine on mouse USV induction are dependent on intrinsic factors relevant to males, and support the notion that frequency-modulated calls are more associated with dopamine function.

Moreover, during male-female interaction, USV production in males did not change after 0.5 mg/kg administration (i.p., p>0.05), whereas USVs were suppressed by higher doses of amphetamine (2 or 6 mg/kg, p<0.01) compared with saline (0 mg/kg, Figure 8A) without visible decrease in sexual interaction. These results suggest that amphetamine could affect mouse USV production, and that the effects of amphetamine on USVs during male-female interaction were relevant to the dose.

In addition, USVs induced by amphetamine (0.5 mg/kg) had a shorter duration (29±1 ms versus 45±2 ms, n = 60, p<0.05), but a much broader bandwidth (30.01±1.62-kHz versus 8.21±1.00, n = 60, p<0.01) than USVs produced during male-female interaction in male mice.

Taken together, these results suggest that 1) amphetamine can induce USVs dose-dependently in single wild-type male mice, but not in female mice; 2) amphetamine-induced USVs may differ in quality from the USVs elicited externally during male-female interaction; 3) M5 muscarinic receptors play an essential role in the induction of USVs; and 4) amphetamine administration also affects USV production during male-female interaction, implying that dopamine activity appears important for USV production in male mice. In addition, we observed that amphetamine (0.2–6 mg/kg) did not induce USVs in C57BL/6 background mice, implying a role of genetic background in amphetamine-induced USV production (data not shown).

### Duration, peak frequency and bandwidth of USVs produced by different male genotypes (Exp. 2–5)

As shown in Table 1, compared with wild-type mice (CD1X129), the peak frequency of USVs in M5 KO mice decreased (50.92±1.28-kHz versus 63.20±0.76-kHz, p<0.01, n = 60 USVs), while the duration and bandwidths remained unchanged. Here, peak frequency was measured by the Avisoft SAS Lab Pro software at the peak intensity of each syllable. Compared with wild-type mice (C57BL/6), in M2 KO mice the peak frequency of USVs was lower (62.62±1.72-kHz versus 67.76±0.75-kHz, p<0.05), but no change was found in duration and bandwidth (p>0.05); in D2 KO mice, a longer duration of USVs was found (63±4ms versus 50±4 ms, p<0.05), but no change was found in frequency or bandwidth (p>0.05). No change was observed in M4 KO mice. These results show that, in addition to the number of USVs emitted by male mice, muscarinic receptors M2 and M5 receptors may play a role in duration, frequency and bandwidth of USVs. In particular, although no change was found in the USV number in D2 KO mice (Figure 7C), D2 receptors played a significant role in inhibiting the duration of USVs (Table 1).

**Table 1:** Duration, peak frequency and bandwidth of the USVs produced by different genotypes of male mice.

Mice	Duration (ms)	Frequency (kHz)	Bandwidth (kHz)
Wild-type (CD1x129)	45±2	63.20±0.76	8.21±1.00
M5 KOs	44±2	50.92±1.28**	10.89±1.50
Wild-type (C57BL/6)	50±4	67.76±0.75	10.21±1.60
M2 KOs	54±4	62.62±1.72*	13.37±1.84
M4 KOs	55±5	67.95±0.76	13.86±2.0
D2 KOs	63±4*	67.78±1.11	12.24±2.20

Duration and peak frequency of USVs were measured automatically according to the protocol introduced in Avisoft SASLab Pro software as described in Materials and Methods. Likewise, the bandwidths of USVs were obtained by measuring the frequency difference of upper and lower bands. A comparison of duration, frequency and bandwidth of USVs between wild-type mice (CD1x129 or C57BL/6) and M2, M4 and M5 muscarinic and D2 dopamine receptor knockout mice was carried out. (Value = Mean±SEM, n = 60 USVs in each group, ^*^p<0.05, ^**^p<0.01, *t*-test).

## Discussion

Male mice produce complex, song-like USVs during sexual behaviour, resembling the complex, song-like USVs induced by exposure to pheromones [14]. In this study, we studied mouse calls under different conditions, and found that: 1) Direct contact of male with female was necessary and sufficient for male USV induction in these experiments. 2) Amphetamine dose-dependently induced USVs in isolated wild-type males, but did not induce USVs in females or M5 KO mice. During male-female sexual interactions, amphetamine increased USVs at 0.5 mg/kg, and decreased the USVs at 2 or 6 mg/kg. 3) Males emitted USVs, which may indicate positive affect; whereas females produced squeaks that may reflect negative affect. 4) There was a characteristic shift of male calls to broken “step-like” USVs with 70-kHz and 40/80 harmonic frequencies and an occurrence of female squeaks after initiation of mounting behavior.

By studying knockout mice, we further found that: 1) USVs were lost in about 75% of M2 KO mice, associated with a loss of sexual interaction. 2) In M5 KO mice, frequency-modulated USVs from male mice were reduced by almost 80%. 3) USVs were not altered reliably in M4 KO mice. 4) M2, M5 and D2 receptors played a role in either duration or peak or bandwidth of USVs.

### USVs induced via odor, sight and direct contact

Male and female mice communicate with each other via odor, sound, sight, vocal communication and/or direct contact. Among them, odor plays an essential role in male-female recognition and sexual behavior. How the rodents coordinate these communicating signals in sexual interactions is unclear. Here, we found that female urine induced male's USVs in a species-dependent way, but strain differences had little effect. These findings suggest two possibilities: 1) Specific recognition via urine odors [4] reduces confusion between species such as mice and rats. 2) Female odors unidirectionally attract male, which respond to females by emitting featured USVs, and females appear to move toward the calls. Under some conditions, female also produce calls in response to aversive stimuli, including those from aggressive males.

Moreover, direct contact with females can facilitate production of male USVs. The transmission via odor and USVs may allow male and female to communicate from farther distances to facilitate copulation. The squeaks emitted by females may be an attempt to fend off the males.

### Dopamine activation and USV production

Dopamine is a neurotransmitter that plays crucial roles in reward, motivation and cognition [51], [52]. Here, we found that the dopamine activator amphetamine induced vigorous USV production at a lower dose (0.5 mg/kg) in wild-type males, but did not induce USVs in either female or M5 KO mice. Dopamine activation at low doses was optimal for USV induction in male mice, but not dependent on the D2 receptor, suggesting that the M5, but not D2, receptor is required for the amphetamine-induced USVs and that the effects of amphetamine on USVs are dependent on intrinsic male factors. Nevertheless, longer duration of USVs was observed in D2 KO mice (see Table 1). This implies a role of D2 receptors in USV quality. Interestingly, compared with the USVs emitted during male-female interaction, a shorter duration and a wider bandwidth were found in amphetamine-induced USVs of wild-type mice. This is the first report of quality change in amphetamine-induced USVs in mice. The mechanisms underlying these changes and the neurobiological meaning remain unknown.

On the other hand, during male-female sexual interactions, amphetamine did not increase USVs at lower doses (0.2 and 0.5 mg/kg) (p>0.05), but suppressed USVs at higher doses (2.0 and 6.0 mg/kg) in males (Figure 8A). Therefore, these results demonstrate that amphetamine injection affected mouse USVs in dose- and sex-dependent ways. Here, induction of USVs by amphetamine is consistent with previous reports in rats [53], [54].

### Deletion of muscarinic or dopamine receptor genes and USVs

Cholinergic neurotransmission plays a crucial role in a variety of functions of the central nervous system, including sensory perception, arousal, motivation, reward and psychosis [24]. M2 muscarinic receptors are expressed widely in the brain, whereas M4 receptors predominate in the basal ganglia and cortex [29]. Although the M5 subtype is the least abundant in the brain, it is located in the midbrain dopamine neurons. In the forebrain, M5 receptors have strong effects on striatal and accumbens dopamine release [21], [25].

In this study, we measured the roles of these muscarinic receptors on USVs in M2, M4 and M5 knockout and wild-type mice. The dramatic reduction of USVs in M2 and M5 KO mice during male-female interactions suggested that both M2 and M5 receptors were crucial for USVs associated with male-female interactions. Further work is needed to clarify the relationship among gene deletion, USV production and sexual behavior in mice. M4 receptors were much less important for the induction of USVs during male-female interaction (see Figure 7B). The localized expression of M5 receptors [28] on ventral tegmental dopamine neurons may underly the importance of M5 receptors in the male-female interaction associated male USVs.

Taking into account of the roles of M5 muscarinic receptor in reward [20], morphine dependence [22], locomotion [28], and its strong effects on striatal and accumbens dopamine release [21], [25], these results support the notion that the abolished USV production in M5 KO mice in response to amphetamine stimulation or the reduced USV production during male-female interaction may result from the role of M5 receptors in controling dopamine release in the forebrain.

Among the 5 dopamine receptors, D2 receptor is perhaps the most thoroughly investigated receptor and has been shown to play an important role in sexual and morphine reward [33]. D2 blockers have been used to treat positive symptoms of schizophrenia for decades [55]. We did not find changes in USV occurrences, however, in D2 KO mice, but we did find a longer duration of USVs. Although D2 receptors play a role in mediating opiate reward, they are only critical in opiate-dependent and withdrawn mice [33], and this effect is also dependent on the genetic background [56]. Moreover, as shown in FoxP2 KO mice [39], the effects of D2 deletion on USV production may not be observable in adult mice, but it might be evident in pups (data not shown). The lengthening of mean USV duration in D2 KO mice suggests that D2 receptors may play a role in inhibiting USV duration.

### Could USVs be used as indices of affective states in mice?

Knutson et al. proposed that USVs in rats could be used to measure anticipatory affective states [57]. Accordingly, long 22-kHz USVs may index a state of negative activation, whereas short, 50-kHz USVs may instead index a state of positive activation [15], [58], [59]. In the present study, the male calls were apparently associated with a state of positive activation since they were detected during male-female interaction as the males approached the females and initiated sexual contact.

USVs could also be observed when the male mice were exposed to fresh female urine. Interestingly, females emitted similar USVs to females after female-female reunion (a positive event in which the females actively approach one another), but not toward males during sexual interactions (in which the females often resisted the males). Therefore, these “approach-related” USVs in both male and female mice may correspond to the 50-kHz calls in rats.

As summarized in Figure S2, the harmonic female calls appeared to be indices of a state of negative activation when females were attacked by or mounted by male mice. This result is consistent with a previous report of aversive squeaks in mice [60]. The call difference between male and female does not appear to exist in rats during male-female interaction [15]. Possibly, the present experiments do not allow females to control or pace male aggression, resulting in a less favorable situation for the females. Activation of the ascending portion of the mesopontine cholinergic system to the medial hypothalamus and septum in rats induces 22-kHz aversive calls while activation of the ascending dopaminergic system induces the positive state with 50-kHz calls in rats [15], [58], [59]. Our findings that USVs were reduced in M5 KO mice are consistent with an involvement of the M5 receptor in the activation of positive calls via the dopamine system.

### Genes and molecular basis of communications in mice

USVs from rodents were once considered by-products of locomotor activity and social signals [34]. However, recent evidence has suggested more complex functions for USVs in rodents. Fifty-kHz positive affective vocalization could be bred selectively in rats [34], or social defeat in rats elicited 22-kHz vocalizations. In mice, male USVs have characteristics of songs [1], [14], and so they may be useful for studying the genetic basis of social communication and emotions [61]. In mouse pups, extensive experiments have been carried out to study the effects of genotypes on USV production [18], [47], [62]–[64]. Here, our experiments using M2, M4, M5 and D2 dopamine receptor KO mice provide direct evidence that USVs produced by mice are likely functional for communication, recognition, sexual interaction, even negative and positive affective states. The dramatic reduction of USVs in M2 and M5 KO mice may suggest a role of these two receptors in USV-mediated male-female recognition during male-female interaction. Both cholinergic and dopaminergic systems are involved in the USVs elicited during male-female interactions, with M2 and M5 muscarinic receptors particularly important in the induction.

What are the pathways by which genes can affect USVs? Ehret proposed that genes can contribute to the perceptual pathways of the nervous system, or to the regulation of emotion and motivation, or directly link to call production [65]. In this study, we found the USV quality was still excellent in M2, M4, M5 muscarinic and the D2 receptors KO mice, implying that motor control in the KO mice is not defective. Furthermore, given the effects of M2, M4, M5 muscarinic receptors and the D2 dopamine receptor in dopamine release [23]–[25], [66], and their varied and wide distribution in the brain [28], [29], we postulate that, these receptors are more likely to contribute to the regulation of emotion and motivation. Our data support the notion that M5 muscarinic receptors may also influence the number of USVs per test period.

### Male USVs, female squeaks and behavior

In addition, USVs could facilitate sexual behavior in female rats [17]. Male USVs may play a role in keeping the female closer to males [16]. Here, we found the coincidence of loss of USVs and sexual behavior in some of the M2 KO mice and the coincidence of characteristic male broken USVs and female squeaks produced during mounting behavior. This finding supports the idea that both male USVs and female squeaks may play a role in facilitating mounting/intromission in mice. More detailed studies, such as USV playback and call blocking, are needed to clarify this point. Overt behaviors, for example, locomotion activity, have been reported previously [24], [28], [67]. In this study, we did not report details about the relationship between USV production and locomotion of individual mice.

In summary, this study described the induction of male USVs which differ from those of female mice during male-female interactions. Male USVs were especially dependent on M2 and M5 muscarinic receptors, but less or not at all on M4 and D2 receptors. The mechanisms underlying changes in duration, frequency, and bandwidth of USVs and the meaning of the changes in different genotypes or in amphetamine-induced USVs are not determined.

## Materials and Methods

### Animals

Generation of homozygous M2, M4 and M5 receptor KO mice [genetic backgrounds: 129J1×CF1 (M2), 129/SvEv×CF1 (M4), and 129/SvEv x CD1] has been described previously [28], [68]–[70]. D2 dopamine receptor KO mice [genetic background: 129J1×C57BL/6], which were generated as described previously [67], were purchased from the Jackson Laboratory (Bar Harbor, Maine, USA). The mice used here were backcrossed on the C57BL/6J background for at least 5 consecutive generations, except M5 KO mice. For each KO strain, the corresponding WT mice were used in parallel as controls. All experiments were performed with 3–5 month old adult male mice. Genotyping was performed by PCR analysis of tail DNA [28], [71]. Adult male and female Wister rats (8 weeks old) were bought from Charles River (St. Constant, Quebec, Canada).

All experimental animals were housed at the Bioscience Support Facility of the University of Toronto and maintained in a light-controlled room (12 hr light/dark cycles) at 21 C. Mice were group-caged with same-sex mice, and food and water were available *ad libitum* except during behavioral testing. All experimental protocols using animals were performed in accordance with the *Guide to the Care and Use of Experimental Animals* (Canada), and approved by the Animal Care Committee of the University of Toronto.

### Apparatus, software and USV analysis

Testing took place in clean rectangular polyethylene cages measuring 29×18×12.5 cm with standard bedding, covered by a metal wire lid. The floor was covered with wood shavings. USVs were recorded with an Ultrasound Detector D 1000X (Pettersson Elektronik AB, Uppsala, Sweden). The condenser microphone was positioned 23 cm above the floor of test cage. All sound equipment used in this study had a flat response to a maximum of 200-kHz unless otherwise indicated. During testing, the occurrence of vocalizations was monitored through headphones with a QMC heterodyne receiver (bat detector) that reduced the frequency of ultrasound into the human auditory range. The ultrasound detector was set to maximum 200-kHz (sampling frequency) enabling it to detect calls through the frequency range of 1 to 200-kHz (in some cases, 1 to 300-kHz). Spectral analyses of the non-transformed ultrasonic recordings were performed using Avisoft SASLab Pro software (Avisoft Bioacoustics, Berlin, Germany) working on Windows XP, which performs a continuous fast-Fourier analysis and displays the results on a screen. The duration, peak frequency and bandwidth of USVs were analyzed according to the Avisoft software manual. The USVs with bandwidth ≤5-kHz is considered as flat USVs, whereas those whose bandwidth being >5-kHz is considered as frequency-modulated USVs. To ensure the accuracy of the data, a certain number of USVs (>60) were counted as flat and frequency-modulated USVs and confirmed by measuring their bandwidths (frequency change). Only a high coefficient ratio (>0.95) for inter-rater-reliability obtained was considered valid data. If the USVs was discontinuous, it was named broken USV. In particular, if USV was broken by a sudden downward step, with a harmonic at the octave, it was named a “broken” USV. A squeak is a human-audible harmonic dense-layered call.

### Experimental Procedures

#### Experiment (Exp.) 1

Effects of fresh urine on USV induction. For this experiment, clean, autoclaved test cages with new bedding (Bed-O'cobs, Andersons., Louisiana, USA) were prepared. The fresh urine was obtained from either male or female mice, rats or humans and could be used immediately or stored at 4°C for further tests. For each test, the urine (0.5–3.0 ml) was added directly onto the bedding of the cage with a male or a female mouse, and then the tests were carried out under dim red light in a quiet room. USVs were recorded with Ultrasound Detector D 1000X, while the behavior was recorded using a video camera (Sony, Japan) for 5 minutes [72]. Eighteen male mice were tested by using urine from 30 mice, 12 female rats, or 2 female humans.

#### Experiment 2

Effects of odor, sight and intimate male-female contact on USV induction. The adult mice to be tested were first habituated in the home cage holding 3–5 mice for 7 days in the test room. On the test day, one female mouse and one male mouse were placed in two separate clean cages and the two cages were gently moved together, followed by 3 min recording. At the end of the recording, the female cage was separated with a dark plate in the middle and the male mouse was placed into one side of it, meanwhile leaving the female mouse on the other side for the second 3 min recording. At the end of the recording, the dark plate was replaced with a transparent plastic plate for the third 3 min recording. Finally, the last 3 min recording was performed after removing the plate and letting the two tested mice contact each other freely. Vocalization recording was started immediately after setup and lasted 3 min for each test with the video recording for further behavior analysis. Twelve male and 6 female mice were tested.

#### Experiment 3

Influence of female hormones on sex-associated male USVs. Instead of normal (intact) female mice, in this experiment, ovariectomized mice were tested with normal naïve male mice. Fourteen to 21 days after ovariectomy, the adult mice to be tested were first habituated in the home cage for 7 days in the test room. On the test day, to test the vocalization of male mice, one ovariectomized female mouse was first placed in a clean standard cage with regular bedding materials, and then a male mouse was added to pair with the female. Vocalization and video recordings were started immediately after the pairing. Twenty male and 10 female were tested.

For mouse ovariectomy, anesthesia was induced by constant isoflurane inspiration. After the onset of anesthesia, the lumbar dorsum was shaved bilaterally and the exposed skin prepared for aseptic surgery (a 10% povidone-iodine scrub followed by a 70% alcohol wipe). For each ovary, a 3/4 cm dorsal flank incision penetrating the abdominal cavity was made. The parovarian fatty tissue was identified and retracted. The exposed ovary and associated oviduct were severed and removed. Hemostasis was achieved by hemostat pressure for 1–2 minutes. Rarely, a ligature (5-0 absorbable suture) around the severed ovarian vasculature was required to maintain hemostasis. The incision was closed using 5-0 nonabsorbable suture in an interrupted pattern.

#### Experiment 4

Influences of M2, M4 and M5 muscarinic receptor and D2 dopamine receptor gene deletion on sexual behaviour-associated male USVs and sexual behavior. The adult mice to be tested were first habituated in a home cage holding 3–5 same-sex mice for 7 days in the colony room. On the test day, to test the vocalization of male mice, one female mouse chosen randomly was first put into a standard clean cage with new bedding materials, and then a male mouse was added to pair with the female. The two mice were then allowed to contact each other. Vocalization recording was started immediately after the pairing and lasted 5 min for each test unless specified. At the same time, video recording by a Sony camera over the testing cage was obtained for further behavior analysis. M2, M4 and M5 muscarinic receptor and D2 dopamine KO mice and wild male and female mice were paired and tested. The obtained data were analyzed with Avisoft SASLab Pro working on Windows XP PC. Seventy mice were tested.

Likewise, for vocalization silencing tests, experiments were carried out similarly with wild-type mice only, but with the mouth of either male or female mouse of a pair simply taped with plastic tape leaving nose open. USV and squeak production were detected as described above.

Regarding sexual behavior, male-chasing-female, male-sniffing-female, and male-mounting-female including intromission were observed. In particular, coincidence of mounting behaviour and production of USVs by males and squeaks by females were analyzed by comparing the sonograms of mouse calls before, during, and after mounting behavior with simultaneously recorded videos. The length of mounting and time period lacking vocalization after the end of mounting behavior were measured.

### Experiment 5

Effects of d-amphetamine sulphate (Sigma, USA) on male-female associated male USVs. The adult mice to be tested were first habituated in a home cage holding 3-5 same-sex mice for 7 days in the colony room. On test day 1, one female mouse was placed in a clean standard cage with new bedding materials, and then a male mouse was injected with 0.2 ml saline and then paired with the female. Vocalization and video recordings were started immediately after the pairing and lasted 5 min for each test. On test day 2, the male mouse was pre-injected with 0.5 mg/kg i,p., amphetamine sulphate, then vocalization and video recordings were performed. Similarly, another group of male mice were used for injection of 0.2 ml saline on day 1, or 1.0 or 2.0 or 6.0 mg/kg amphetamine on day 2. For testing amphetamine-induced spontaneous USVs, male and female mice were tested independently at doses of 0.1, 0.2, 0.5, 2.0, and 6.0 mg/kg. Likewise, vocalization and video recordings were carried out as described above.

To test the effects of amphetamine on the USVs emitted during male-female interaction, similar protocol was used, but the male mice were injected with a varied doses of amphetamine before male-female pairing. Sixty six males and 12 females were tested.

### Statistical analyses

Unless specified, results were expressed as mean±SEM. Statistical comparisons were performed with Student's *t*-test. A two-way ANOVA (SPSS version 12) was tested for wild-type and mutant mice. To compare performance between genotypes, the Student's t-test with Bonferroni correction was used. In all cases, *p*<0.05 was considered to be statistically significant.

## Supporting Information

Figure S1 Female mouse-emitted harmonic dense-layered calls (varied frequencies' calls, 10- to100-kHz, upper panel). Two female-derived calls overlapped with two frequency-modulated male USVs.
(0.07 MB PPT)

Figure S2 Summary of calls induced by male-female interaction. First, calls were classified into male USVs and female calls. In females (right arrows), there were only audible harmonic dense-layered calls related to aversive stimuli from males. Male USVs (left arrows) were more complex and divided into different categories according to the complexity of the frequency modulation, or continuity of the calls. Male USVs reflect positive emotional states in male mice whereas squeaks may serve as indices of negative state in female mice.
(0.02 MB PPT)

Table S1 USVs were not detected 4 minutes after 10 drops of fresh urine added into the cage.
(0.03 MB PPT)

Table S2 No USVs were detected when males were silenced in 5 male-female pairs; but when females were silenced, USVs were detected in 4 pairs.
(0.03 MB PPT)

Table S3 USVs were not detected in 6 out of 8 M2 KO mice, correlated with a loss of sexual interaction (male-chasing-female, male-sniffing-female, and male-mounting-female) between male and female mice. USVs were detected on all wild-type and M4 KO mice tested.
(0.03 MB PPT)
