# Supplementary figures and images for: Ultrasonic Vocalizations Induced by Sex and Amphetamine in M2, M4, M5 Muscarinic and D2 Dopamine Receptor Knockout Mice
Source: PLoS One. 2008 Apr 2;3(4):e1893. doi: 10.1371/journal.pone.0001893 (PMC2268741; doi:10.1371/journal.pone.0001893)

## Slide 1
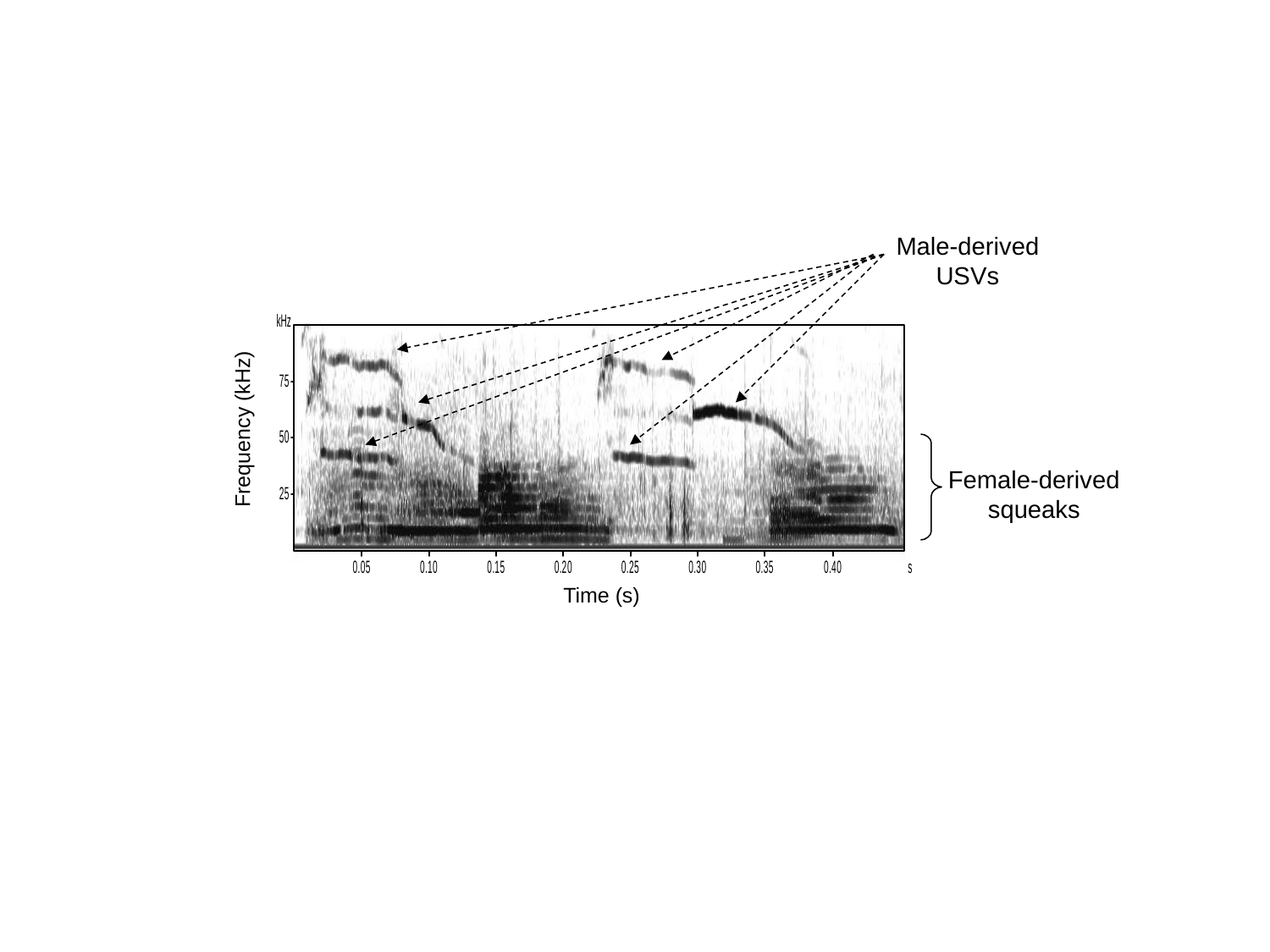

Male-derived
USVs
Frequency (kHz)
Female-derived
squeaks
Time (s)

Supplement: Figure S1 — Female mouse-emitted harmonic dense-layered calls (varied frequencies' calls, 10- to100-kHz, upper panel). Two female-derived calls overlapped with two frequency-modulated male USVs. (0.07 MB PPT) [file pone.0001893.s001.ppt]

## Slide 1
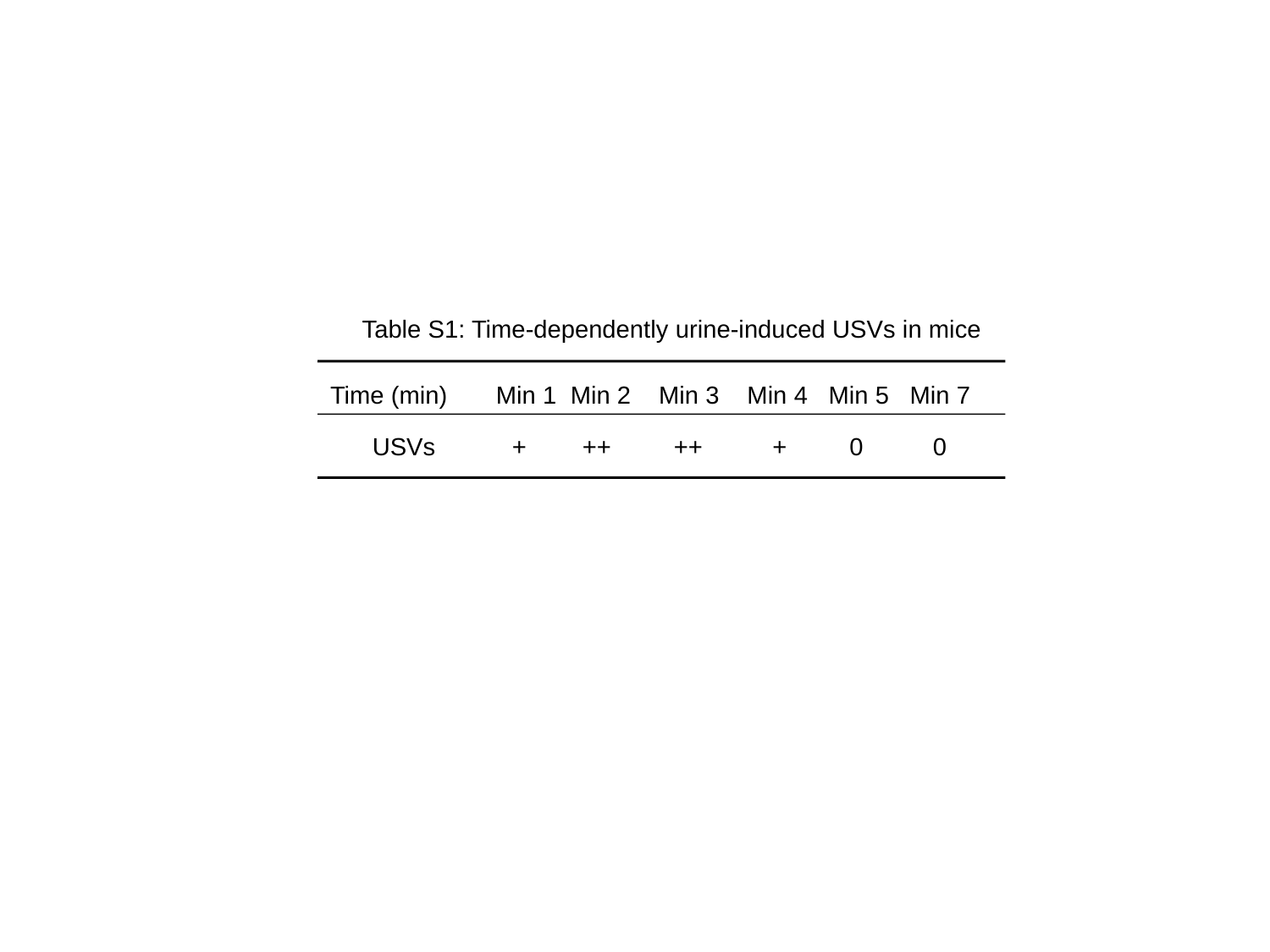

Table S1: Time-dependently urine-induced USVs in mice
Time (min) Min 1 Min 2 Min 3 Min 4 Min 5 Min 7
USVs + ++ ++ + 0 0

Supplement: Table S1 — USVs were not detected 4 minutes after 10 drops of fresh urine added into the cage. (0.03 MB PPT) [file pone.0001893.s003.ppt]
