# Supplementary material for: Ultrasonic Vocalizations Induced by Sex and Amphetamine in M2, M4, M5 Muscarinic and D2 Dopamine Receptor Knockout Mice
Source: PLoS One. 2008 Apr 2;3(4):e1893. doi: 10.1371/journal.pone.0001893 (PMC2268741; doi:10.1371/journal.pone.0001893)
Supplement: Figure S2 — Summary of calls induced by male-female interaction. First, calls were classified into male USVs and female calls. In females (right arrows), there were only audible harmonic dense-layered calls related to aversive stimuli from males. Male USVs (left arrows) were more complex and divided into different categories according to the complexity of the frequency modulation, or continuity of the calls. Male USVs reflect positive emotional states in male mice whereas squeaks may serve as indices of negative state in female mice. (0.02 MB PPT) [file pone.0001893.s002.ppt]

## Slide 1
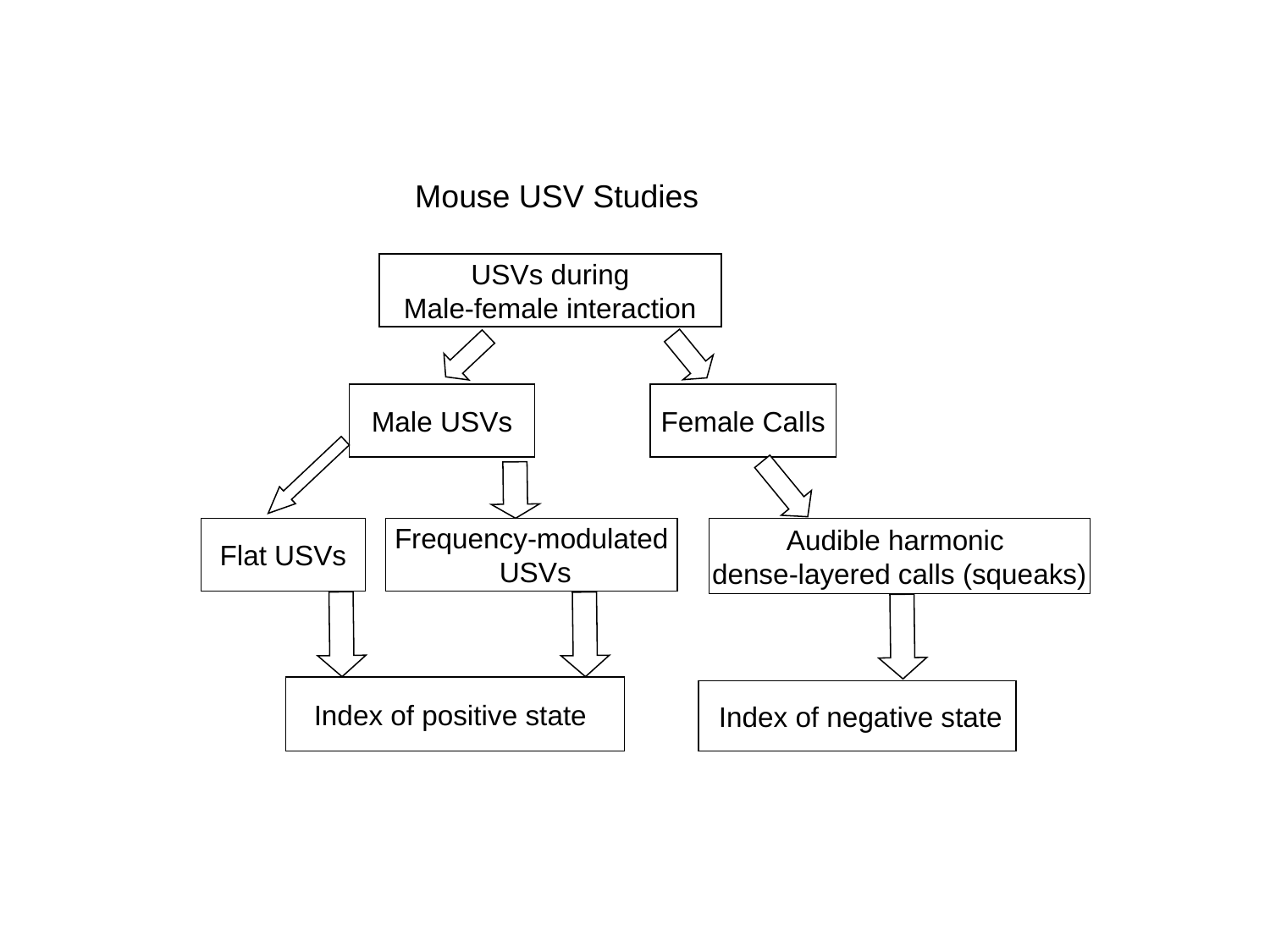

Mouse USV Studies
USVs during
Male-female interaction
Male USVs
Female Calls
Flat USVs
Frequency-modulated
 USVs
Audible harmonic
dense-layered calls (squeaks)
 Index of positive state
 Index of negative state
