# Supplementary material for: Ultrasonic Vocalizations Induced by Sex and Amphetamine in M2, M4, M5 Muscarinic and D2 Dopamine Receptor Knockout Mice
Source: PLoS One. 2008 Apr 2;3(4):e1893. doi: 10.1371/journal.pone.0001893 (PMC2268741; doi:10.1371/journal.pone.0001893)
Supplement: Table S2 — No USVs were detected when males were silenced in 5 male-female pairs; but when females were silenced, USVs were detected in 4 pairs. (0.03 MB PPT) [file pone.0001893.s004.ppt]

## Slide 1
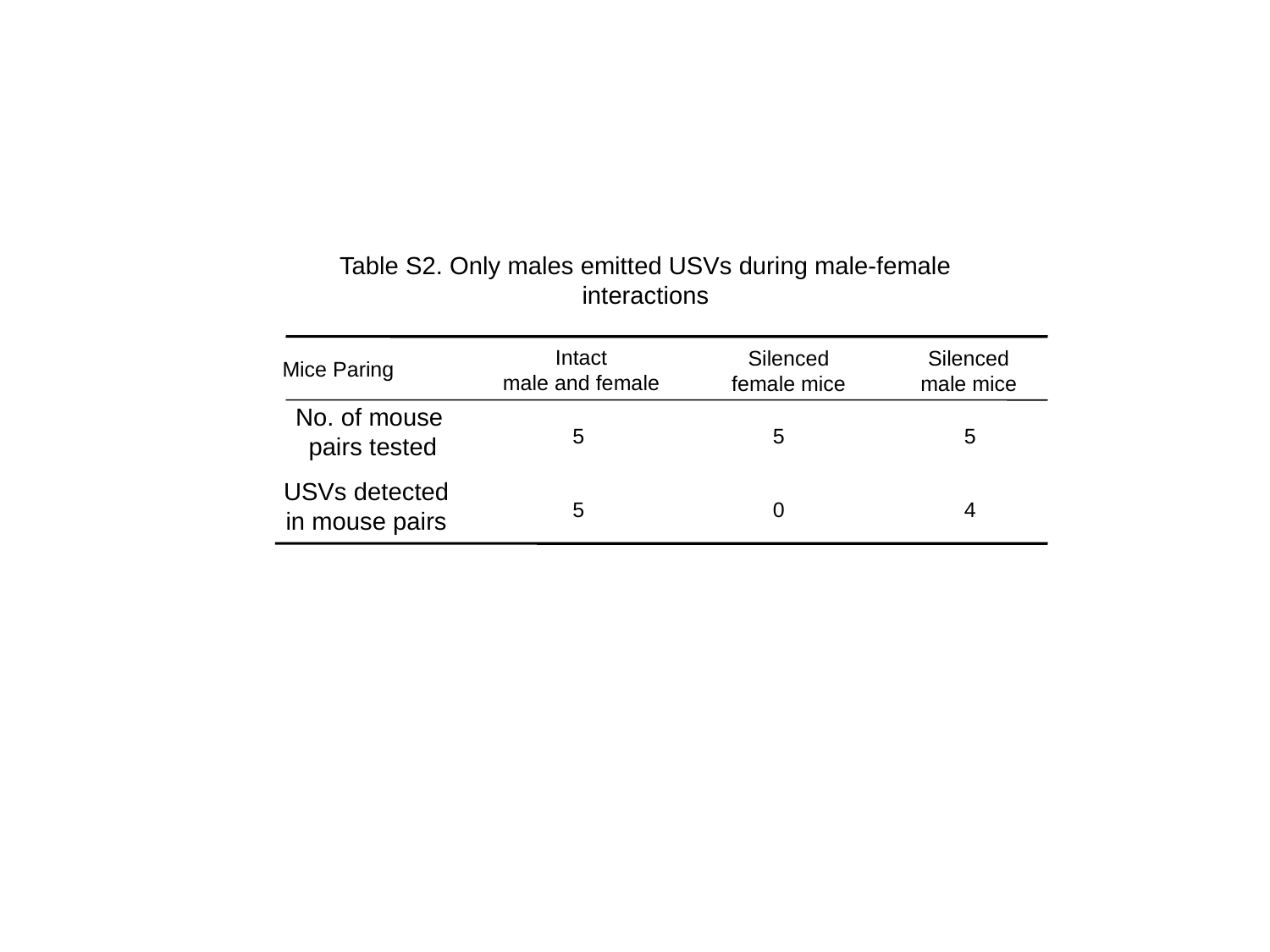

Table S2. Only males emitted USVs during male-female interactions
Intact
male and female
Silenced
female mice
Silenced
male mice
Mice Paring
No. of mouse
pairs tested
5
5
5
 USVs detected
in mouse pairs
5
0
4
