# Supplementary material for: Ultrasonic Vocalizations Induced by Sex and Amphetamine in M2, M4, M5 Muscarinic and D2 Dopamine Receptor Knockout Mice
Source: PLoS One. 2008 Apr 2;3(4):e1893. doi: 10.1371/journal.pone.0001893 (PMC2268741; doi:10.1371/journal.pone.0001893)
Supplement: Table S3 — USVs were not detected in 6 out of 8 M2 KO mice, correlated with a loss of sexual interaction (male-chasing-female, male-sniffing-female, and male-mounting-female) between male and female mice. USVs were detected on all wild-type and M4 KO mice tested. (0.03 MB PPT) [file pone.0001893.s005.ppt]

## Slide 1
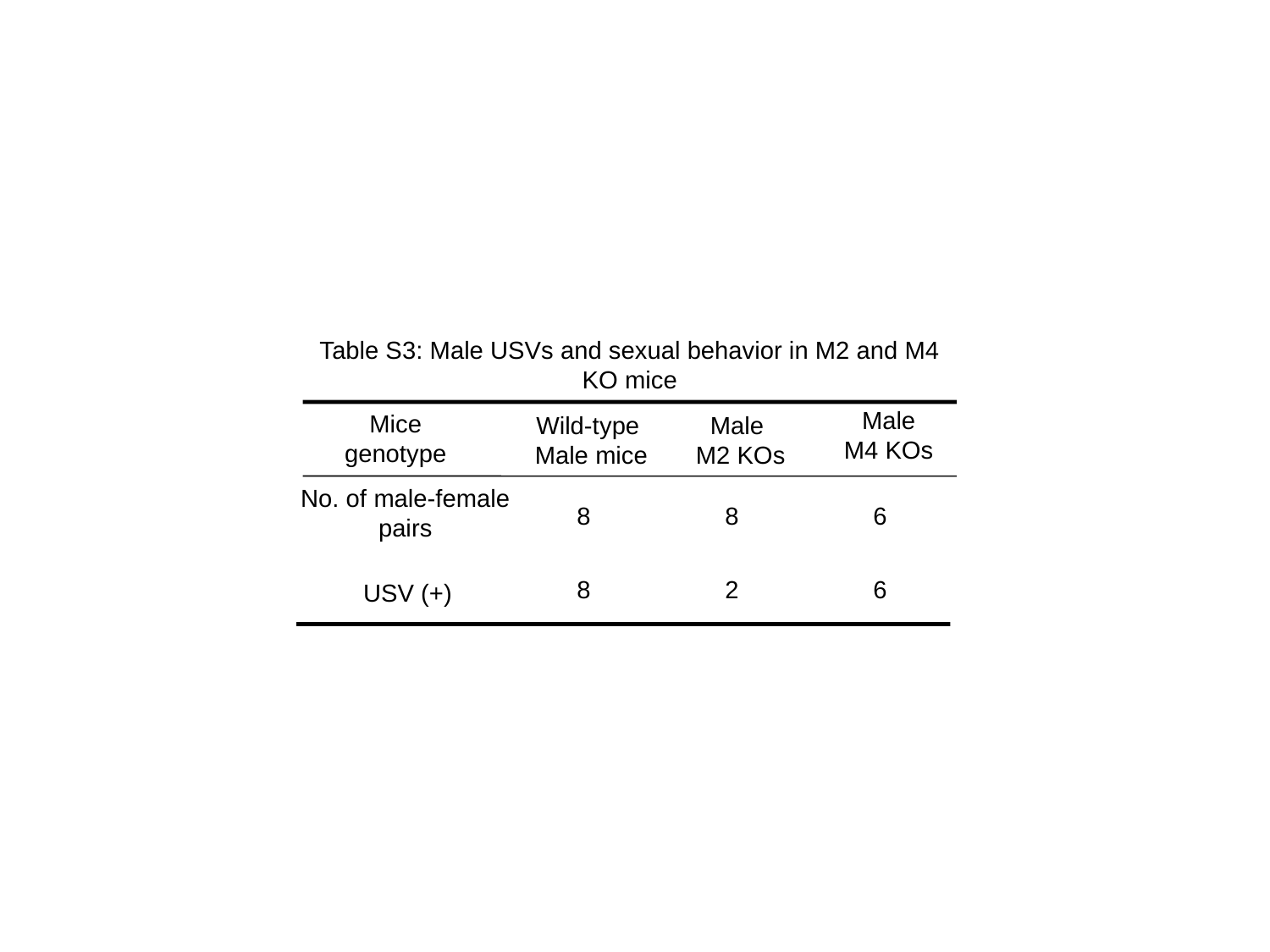

Table S3: Male USVs and sexual behavior in M2 and M4 KO mice
Male
M4 KOs
Mice
genotype
Wild-type
Male mice
Male
M2 KOs
No. of male-female
pairs
8
8
6
8
2
6
 USV (+)
